# The accelerated aging model reveals critical mechanisms of late-onset Parkinson’s disease

**DOI:** 10.1186/s13040-020-00215-w

**Published:** 2020-06-10

**Authors:** Shiyan Li, Hongxin Liu, Shiyu Bian, Xianzheng Sha, Yixue Li, Yin Wang

**Affiliations:** 1 Department of Biomedical Engineering, School of Fundamental Sciences, China Medical University, Shenyang, 110122 Liaoning Province China; 2 China Medical University, The Queen’s University of Belfast Joint College, China Medical University, Shenyang, 110122 Liaoning Province China; 3 Bio-Med Big Data Center, Key Laboratory of Computational Biology, CAS-MPG Partner Institute for Computational Biology, Shanghai Institute of Nutrition and Health, Shanghai Institutes for Biological Sciences, University of Chinese Academy of Sciences, Chinese Academy of Science, Shanghai, 200031 China; 4 School of Life Sciences and Biotechnology, Shanghai Jiao Tong University, Shanghai, 200240 China; 5 Collaborative Innovation Center of Genetics and Development, Fudan University, Shanghai, 200433 China; 6 Shanghai Center for Bioinformation Technology, Shanghai Academy of Science and Technology, Shanghai, 201203 China; 7 Tumor Etiology and Screening Department of Cancer Institute and General Surgery, The First Affiliated Hospital of China Medical University, 155# North Nanjing Street, Heping District, Shenyang City, 110001 Liaoning Province China

**Keywords:** Late-onset Parkinson’s disease, Aging acceleration, Network analysis

## Abstract

**Background:**

Late-onset Parkinson’s disease (LOPD) is a common neurodegenerative disorder and lacks disease-modifying treatments, attracting major attentions as the aggravating trend of aging population. There were numerous evidences supported that accelerated aging was the primary risk factor for LOPD, thus pointed out that the mechanisms of PD should be revealed thoroughly based on aging acceleration. However, how PD was triggered by accelerated aging remained unclear and the systematic prediction model was needed to study the mechanisms of PD.

**Results:**

In this paper, an improved PD predictor was presented by comparing with the normal aging process, and both aging and PD markers were identified herein using machine learning methods. Based on the aging scores, the aging acceleration network was constructed thereby, where the enrichment analysis shed light on key characteristics of LOPD. As a result, dysregulated energy metabolisms, the cell apoptosis, neuroinflammation and the ion imbalances were identified as crucial factors linking accelerated aging and PD coordinately, along with dysfunctions in the immune system.

**Conclusions:**

In short, mechanisms between aging and LOPD were integrated by our computational pipeline.

## Introduction

As the second most common neurodegenerative syndrome (only after Alzheimer’s disease), Parkinson’s disease (PD, namely paralysis agitans), affects 1% of the worldwide population over the age of 60 years [1]. It is characterized by the motor (i.e. resting tremor, movement slowness, rigidity, and postural instability) and nonmotor symptoms (i.e. hyposmia, sleep disorders, autonomic dysfunction, neuropsychiatric alterations, and sensory symptoms) [1]. The motor and non-motor disorders caused by dopamine deficiency severely affect the life quality of the aging population. But up till now, there is not available curative and disease-modifying therapies for PD [2]. With the prevalence of PD increasing, it is emerging as a socio-economic burden and a serious problem for the public health [2].

**Fig. 1:**
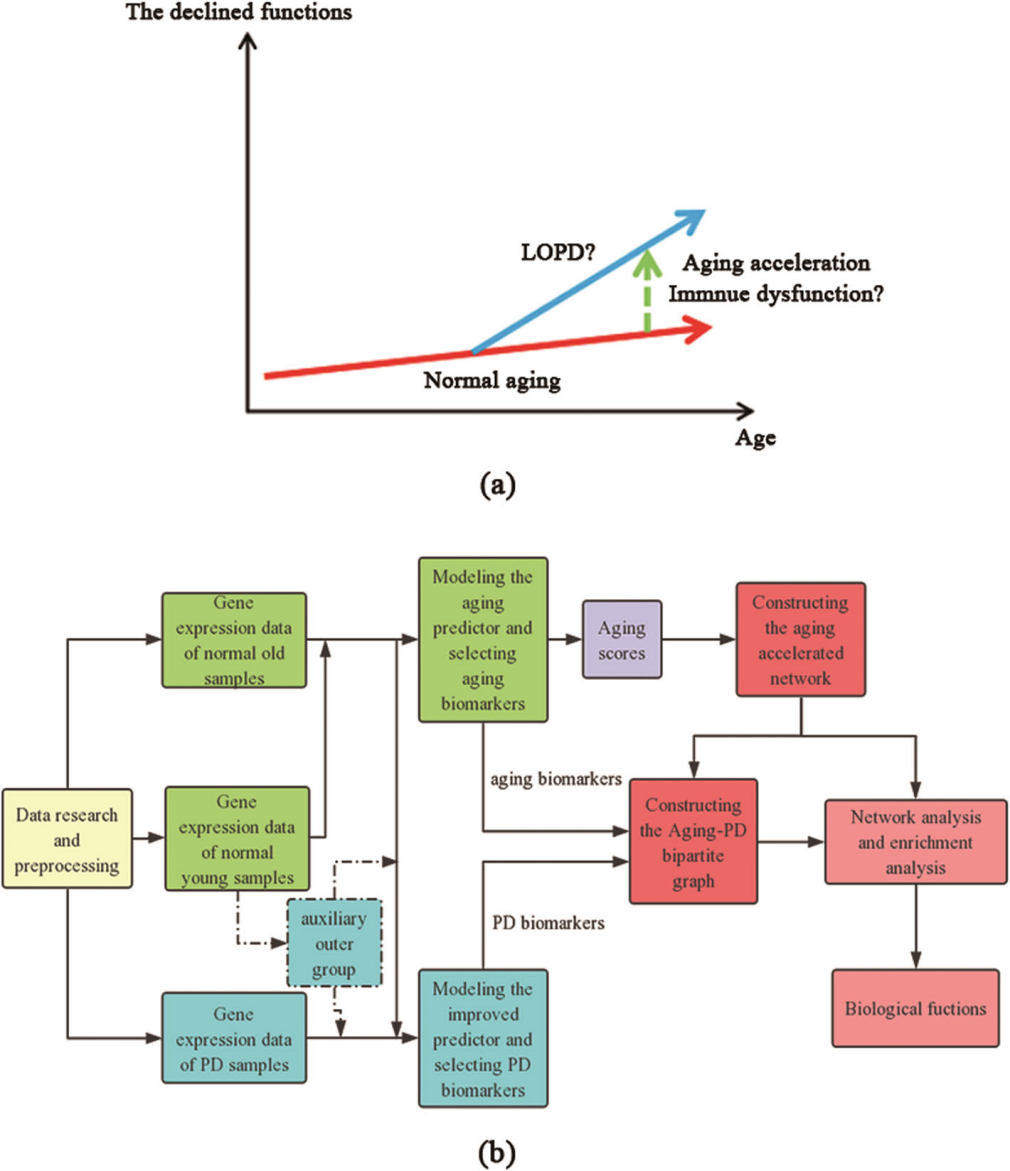
**a** The mechanism hypothesis diagram of LOPD; **b** The workflow in our work

There were abundant epidemiological evidences supported that aging was the primary risk factor for Late-onset Parkinson’s disease (LOPD) [3]. The prevalence of LOPD increased steadily with age [4], indicating aged brains were vulnerable to PD compared with young brains. Thus, the relationship between LOPD and aging was inseparable. First, there were some similar behavior changes between the healthy elderly people and the PD patients, such as slowness of motion, postural instability and cognitive deficits [5]. Second, the age at onset of PD significantly influenced the phenotype and progression of the disease [3]. In other words, age-related factors could predispose old individuals to develop this common neurodegenerative disease. Impaired mitochondrial functions [1, 5], the cell apoptosis [6, 7], and the chronic inflammatory process [8] showed commonalities in both PD and normal aged people. Third, the loss of substantia nigra (SN) neurons between normal aging and PD was significant, where the loss of neurons was 28.3% in older controls compared with younger controls, and the loss of pigmented neurons in PD was 73% compared with older controls [9]. It is noteworthy that SN neurons were the most susceptible to degeneration of PD [3]. Additionally, LOPD was often considered as a result of aging acceleration [5]. Therefore, to obtain a comprehensive understanding between aging and PD, we made a presumption that the onset of PD was induced by accelerated aging through a series of mechanisms (i.e. dysfunctions in the immune system, Fig. 1a).

Several studies have focused on the molecular mechanisms of PD development and in connection with the aging process [10]. Nevertheless, it is still insufficient to uncover the underlying relationship between PD and aging acceleration based on systematic models, which were informative to study PD. Fortunately, it has been demonstrated that machine learning techniques in correctly classifying healthy and PD patients performed well [11], thus predictive models based on genetic datasets were used to identify potential biomarkers, which was powerful to trial design and evaluation, as well as diagnosis and treatment of PD [12]. Recently, different approaches have been proposed for accurate characterization and diagnosis of PD (i.e. the voice disorder, gait, age and so on) by statistical analyses and machine learning methods [13, 14]. However, these results neither deeply revealed the connection between aging and PD, nor indicated the mechanisms of LOPD regulated by aging acceleration. Hence, systemic models were urgent to understand the trigger mechanisms of PD during aging acceleration (Fig. 1a), still needing to be improved by machine learning and network methods.

In the manuscript, to explore the disturbance of biological functions during the aging process, a computational pipeline was developed on the basis of both normal and PD transcriptional profiles (Fig. 1b). (1) The improved PD predictor (by comparing with normal aging) was contributed to identify risk biomarkers, as well as the aging predictor, where the kNN algorithm was used to capture data features from the complex non-linear characteristics of aging and neurodegenerative diseases, respectively [15]. (2) The aging scores were further summarized based on aging markers. (3) The aging accelerated network was constructed based on both correlation and partial correlation of each gene pair. (4) Both network analysis and enrichment analysis were also conducted to identify potential mechanisms of LOPD regulated by aging acceleration. In conclusion, our pipeline was designed to investigate the connection between accelerated aging and LOPD at system level.

## Results

### Modeling the aging predictor and identifying aging markers

**Fig. 2:**
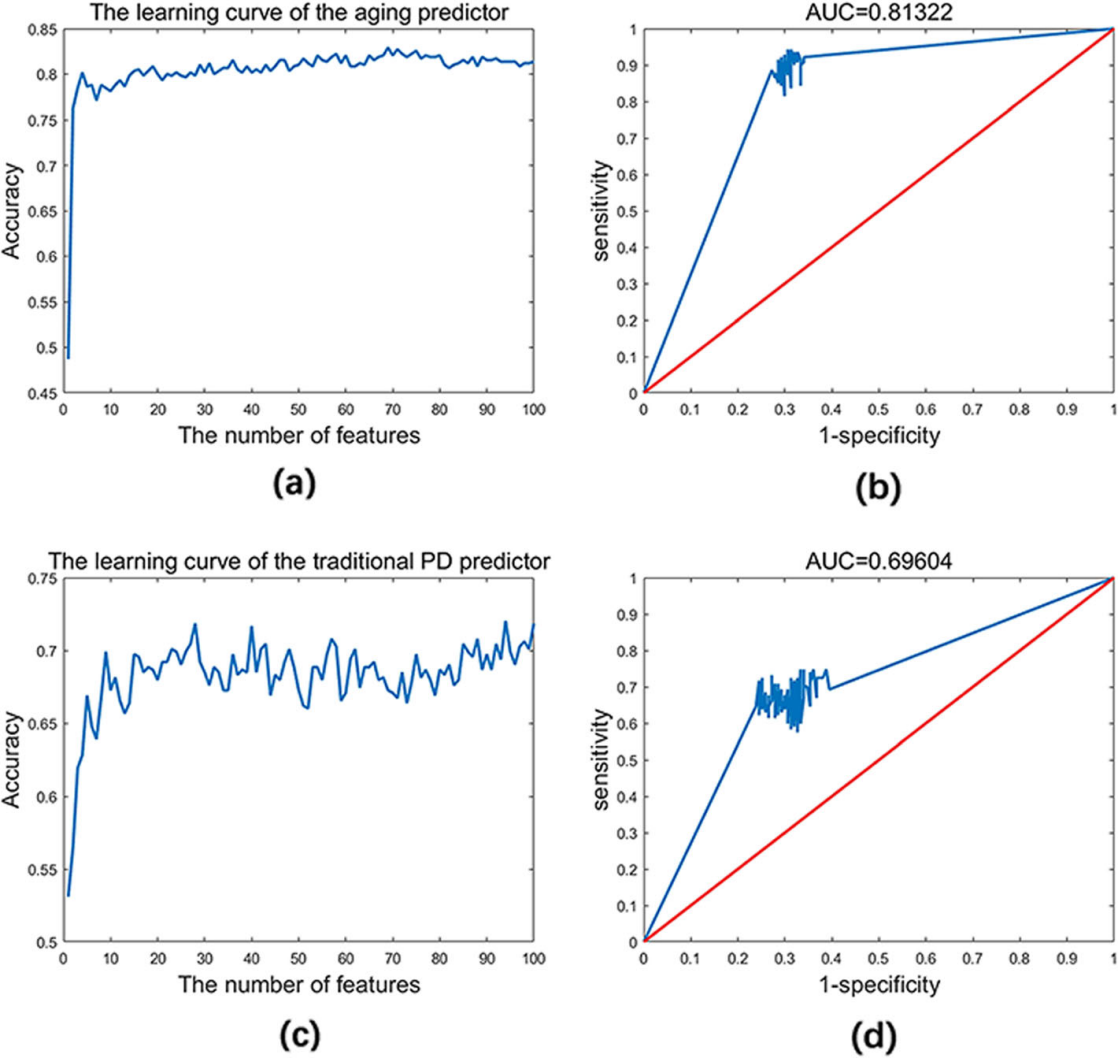
The learning curve and ROC curve in the aging predictor and improved PD predictor. **a** The learning curve of the aging predictor; **b** The ROC curve of the aging predictor; **c** The learning curve of the improved predictor; **d** The ROC curve of the improved PD predictor

The total gene expression profile including 13,883 genes (Text S1) from six GSE profiles was analyzed. The details of the young samples and the old samples for the training dataset and test dataset were shown in Text S2. We utilized Pearson correlation coefficient and k-Nearest Neighbors (kNN, k = 5 and the cosine distance) to model the aging predictor and selected the optimal one based on the 10-fold cross-validation. The learning curve of the training dataset was shown in Fig. 2a. As a result, the top 69 genes were identified as the aging markers (Table S1). The accuracy in the test dataset was 0.8109. The receiver operating characteristic (ROC) curve was shown in Fig. 2b, and the area under the ROC curve (AUC) was 0.81322. Obviously, these related results indicated the reliability of the optimal aging predictor.

It was noteworthy that the selected aging biomarkers were of great significance. For example, RBMS1 was the most relevant gene (with the highest ranking) among the 69 aging markers. As previously reported, RBMS1 encoded a cell cycle suppressor, which bound to an enhancer element of MYC [16]. The transcription protein of RBMS1 was implicated in DNA replication, transcription, and cell apoptosis [17]. As a result, these dysfunctional cell activities might lead to the accumulation of cellular senescence and aging [18]. In short, RBMS1 was closely related to the aging process.

### Modeling the improved PD predictor and identifying PD markers

In order to better understand how LOPD was triggered in the context of aging acceleration, an improved PD predictor was constructed by discriminating PD and normal aged groups compared with the healthy young group. The details of the number of aged normal and PD samples in both training and test dataset were listed in Text S2. The importance of 13,883 genes was ranked by the absolute values of Pearson correlation coefficients, and the k-NN algorithm (k = 5 with the cosine distance) was used to model the PD predictor. Then the optimal model as well as PD markers were selected by the 10-fold cross-validation. The top 8 markers were considered as key PD markers (Table S2). And the learning curve was shown in Fig. 2c. For the stability of the selected model, it was validated in the total test dataset, the accuracy was 0.7039. The ROC curve for the PD predictor was shown in Fig. 2d and the AUC value was close to 0.7. To evaluate the accuracy of the improved PD predictor, we performed the same operation with the original gene profiles. The results showed that the prediction accuracy of the total test dataset was 0.6882 (Table 1). The related curves and biomarkers were also shown in Figure S1 and Table S3. In summary, compared with traditional PD predictor, the improved PD predictor performed more effectively.

**Table 1:** The accuracy of the training dataset and test dataset for the aging predictor, the improved predictor as well as the traditional predictor

	The accuracy for training dataset	The accuracy for test dataset
The aging predictor	0.8291	0.8109
The improved PD predictor	0.7274	0.7039
The traditional PD predictor	0.7204	0.6882

It was intriguing that ADD2 was identified as the top biomarker in the improved PD predictor. ADD2 was abundant in erythrocytes and brain tissue [19], and its proteins were associated with the assembly of spectrin-actin network and signal transduction pathways by interacting with protein kinase C-dependent and calcium/calmodulin-dependent pathways [20]. It has been reported that a delayed calcium efflux and mitochondrial Ca^2+^ overload may induce cell deaths and neuronal loss in neurodegenerative disorders [21]. Accordingly, ADD2 probably contributed to the PD pathology and was involved in the aging process by regulating the mitochondrial functions.

### The aging acceleration pattern between PD and normal aged samples

**Fig. 3:**
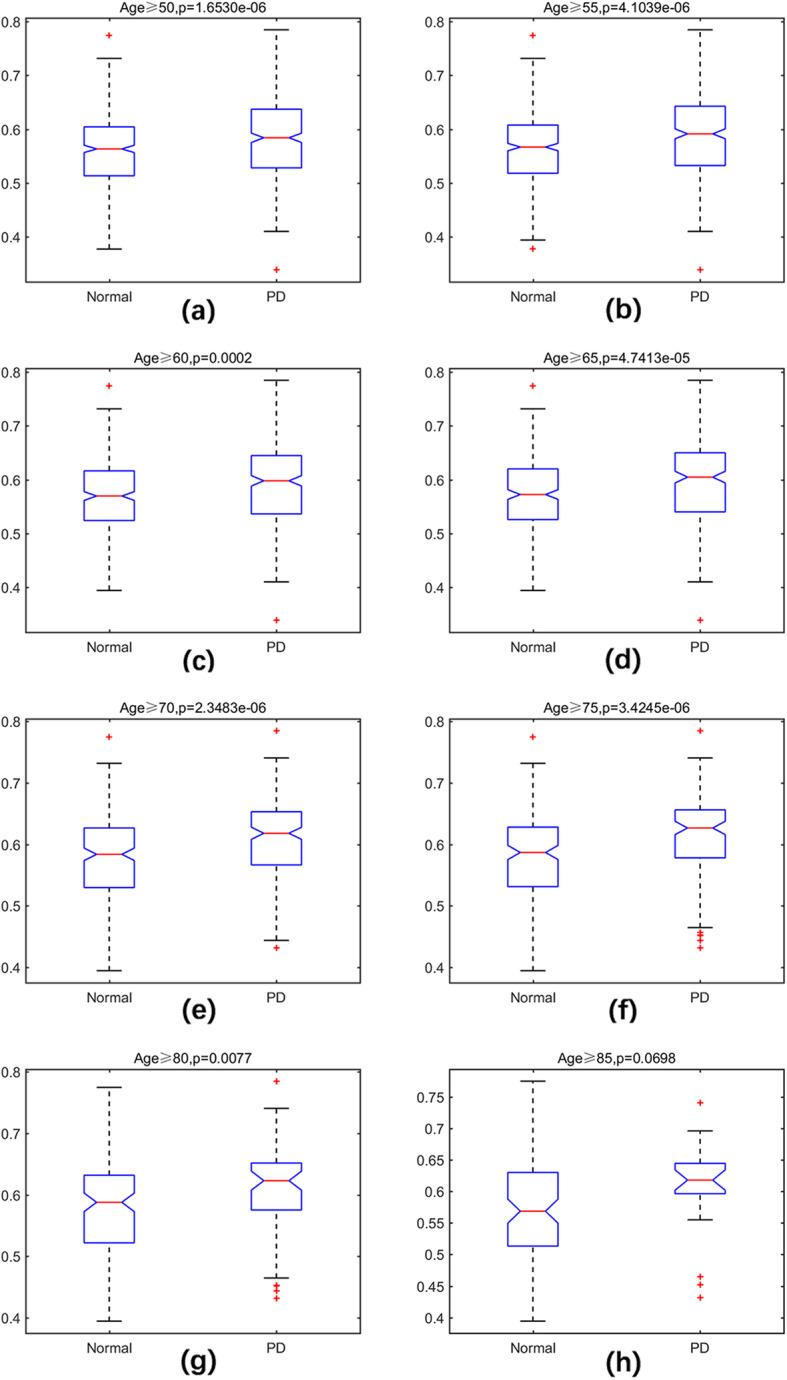
The Kruskal-Wallis test of aging scores for different age groups in the normal samples and PD samples. **a** Age ≥ 50; **b** Age ≥ 55; **c** Age ≥ 60; **d** Age ≥ 65; **e** Age ≥ 70; **f** Age ≥ 75; **g** Age ≥ 80; **h** Age ≥ 85

To investigate the aging acceleration in LOPD, the aging scores were calculated by regressing the transformated age (using the sigmoid function) based on the aging markers. For distinct age groups, the median and mean of the chronological age and aging scores were shown in Table 2. There were increasing trends of the aging scores in both PD and normal aged samples, along with the chronological age. Apart from this, the aging scores of PD samples were always higher than normal samples within the same age group, although the median and mean of chronological age were lower. The results indicated that LOPD samples presented aging acceleration compared with normal aged samples. Therefore, when judging the level of aging, the chronological age did not fully reflect the aging rate of the body, but the aging score seemed to be more credible. In addition, the Kruskal-Wallis test was utilized to test the differences between LOPD and control samples. The results in Fig. 3 indicated that aging scores increased roughly with age, and almost all of the aging scores between PD and normal groups were significantly different (*p* < 0.05), revealing the accelerated aging patterns in most age groups of LOPD. Generally, the aging scores that used to evaluate the accelerated aging patterns in LOPD were of feasibility.

**Table 2:** The chronological age and aging scores of PD and control in different age groups

Age	The median age in PD	The median age in control	The mean age in PD	The mean age in control	The median aging score in PD	The median aging score in control	The mean aging score in PD	The mean aging score in control
≥50	69	71	68.82	71.21	0.584265	0.563570	0.582653	0.560505
≥55	70	72	70.39	72.65	0.591580	0.567050	0.585589	0.564299
≥60	72	76	72.43	77.02	0.598085	0.569840	0.588650	0.570399
≥65	75	79	74.79	79.84	0.604960	0.572590	0.594936	0.572388
≥70	76	82	77.20	82.30	0.617940	0.583710	0.606210	0.577525
≥75	78.5	85	79.90	85.26	0.626575	0.586700	0.614572	0.580568
≥80	84	87	83.68	88.02	0.622935	0.587725	0.610096	0.580391
≥85	87	90	86.77	90.57	0.617940	0.568660	0.603662	0.574285

**Table 3:** Enrichment analysis results with the most numerous enriched paths (the minimum p-value and FDR)

Name	*P*-value	FDR
KEGG_PPAR_SIGNALING_PATHWAY (3 paths)	6.06e-05	0.0113
	2.03e-05	0.0038
	6.0624e-05	0.0113
GO_ALPHA_BETA_T_CELL_DIFFERENTIATION (3 paths, GO:0046632)	1.21e-04	0.0744
	4.06e-05	0.0996
	4.06e-05	0.0498
GO_POSITIVE_T_CELL_SELECTION (2 paths, GO:0043368)	5.4793e-06	0.0395
	5.4793e-06	0.0298
GO_T_CELL_SELECTION (2 paths, GO: 0045058)	1.0741e-05	0.0395
	1.0741e-05	0.0298

### The aging acceleration network revealed potential biological functions

To integrate aging and LOPD markers, the aging acceleration networks were constructed in the training data and the test data based on the aging scores (as mentioned in “Materials and Methods”), respectively. Further, the Fisher’s exact test was carried out to calculate the similarity between these two networks, the result showed *p*-value was very closed to 0. The result indicated that our method was of reliability.

**Fig. 4:**
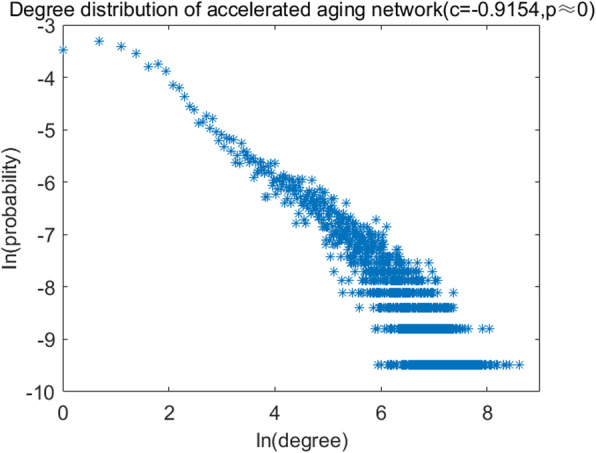
The degree distribution of the accelerated aging network. The Pearson correlation coefficient is − 0.9154 and *p*-value is close to 0

To validate the scale-free characteristic, the curve of node degree distribution was shown in Fig. 4, where the logarithmic transformation was performed in both degrees and corresponding probabilities to test the power-law distribution. As a result, the Pearson correlation coefficient was − 0.9154 (p ≈ 0). It illustrated that the aging acceleration network was in accordance with the scale-free characteristic (there was a negative correlation between the degree and the frequency, where only a small ratio of genes were with high degrees).

Considering key functions of the nodes with maximum node degrees, the gene nodes were ranked based on the degrees. SLC37A1 was with the maximum degree (=5465). SLC37A1 is a Pi-linked glucose-6-phosphate (G6P) antiporter and probably catalyzes both homologous (Pi/Pi) and heterologous (G6P/Pi) exchanges [22]. Furthermore, it has been reported that SLC37A1 is related to transport glycerol-3-phosphate (G3P), where the disturbance of ATP would affect these critical functions in brain [23]. In short, SLC37A1 may play an important role between aging and PD through the mitochondrial function.

### Underlying PD mechanisms based on the enrichment analysis in the aging acceleration network

**Fig. 5:**
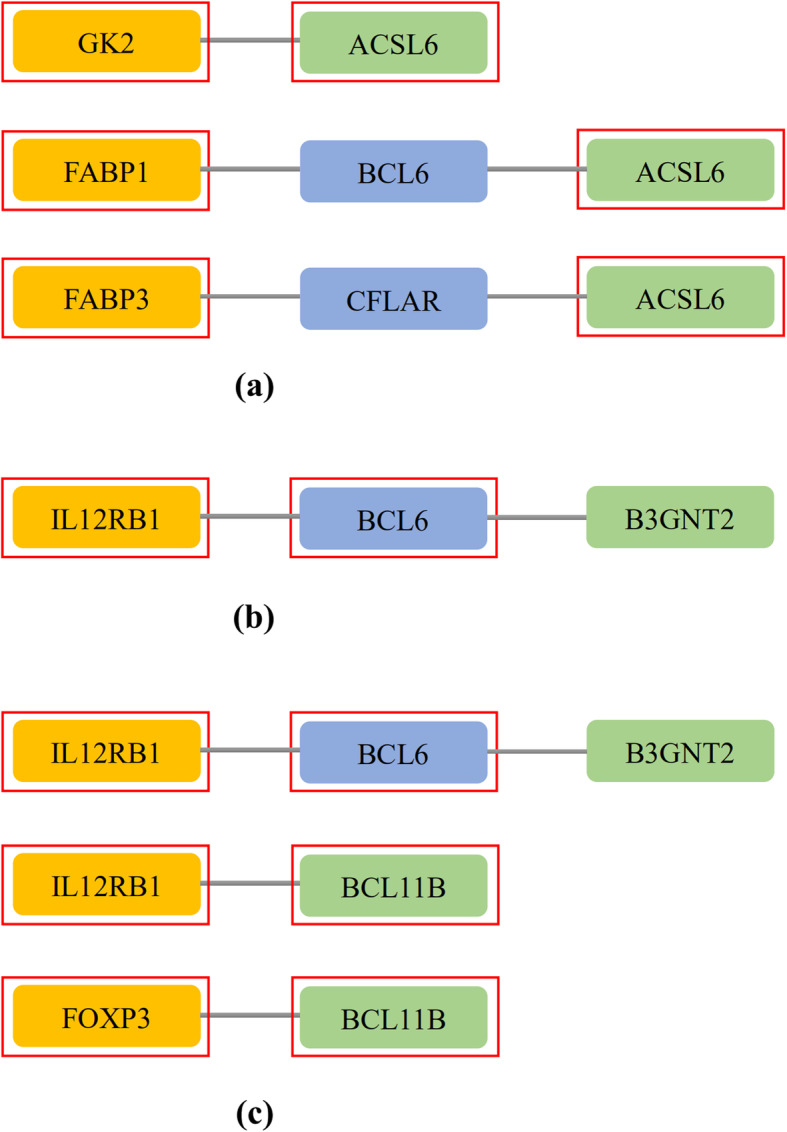
The shortest paths for enrichment analysis of KEGG and BP. **a** The maximum number of enriched paths for KEGG pathway (PPAR signaling pathway, *p* = 6.0624e-05, FDR = 0.0113; *p* = 2.0267e-05, FDR = 0.0038; *p* = 6.0624e-05, FDR = 0.0113); **b** The most significant BP path (Immunological memory formation process, GO:0090715, *p* = 8.7146e-07, FDR = 0.0051); **c** The maximum number of enriched paths for BP pathway (Alpha-Beta T cell differentiation, GO:0046632, *p* = 1.2141e-04, FDR = 0.0744; *p* = 4.0638e-05, FDR = 0.0996; *p* = 4.0638e-05, FDR = 0.0498). The yellow nodes represent the aging biomarkers, the blue nodes represent the genes connecting aging biomarkers and PD biomarkers, the green nodes represent the PD biomarkers, and the genes in the red square frames coincide with those genes in the enriched functions. “PPAR*”: the PPAR signaling pathway

To investigate the relationship between aging and LOPD, the Aging-PD shortest paths were identified based on aging acceleration network. Then the enrichment analysis (i.e. Kyoto Encyclopedia of Genes and Genomes (KEGG) pathway and Biological Process (BP) terms, shown in Table 3) was performed based on each shortest path, respectively. The most significant KEGG pathway was the PPAR signaling pathway (*p* = 2.0267e-05, FDR = 0.0038, Fig. 5a). Interestingly, it was also with the maximum number of enriched paths (3 paths). There were three isotypes of PPAR: PPARα, PPARβ/δ and PPARγ, and their functions were related to the immunotolerance, lipid/glucose metabolism, angiogenesis and the inflammatory response [24]. PPARγ was critical to neuroprotective and anti-inflammatory responses, thus reduced inflammation-driven neuronal damages [25], cell deaths and the progression of neurodegeneration [26]. A current research even has indicated that the PPAR signaling pathway was as the potential target of neuroprotection [27]. Moreover, other results also showed that PPARγ was a critical therapeutic target in Parkinson’s disease, by regulating fatty acid oxidation, immune responses and the mitochondrial function [28]. In short, the PPAR signaling pathway was vital to LOPD.

The immunological memory formation process (GO:0090715) was the most enriched BP term (*p* = 8.7146e-07, FDR = 0.0051, Fig. 5b). Memory cells were widely associated with aging. The percentage of memory T cells were with an increasing trend with age, indicating the importance of memory cells during the aging process [29]. Besides, the treatment of CD4+ T cells in PD patients has been drawn more attentions [29]. To sum up, the functions of the immunological memory linking aging acceleration and PD were confirmed thereby.

The alpha and beta T cell differentiation (GO:0046632) was the BP term enriched in most shortest “aging-PD” paths (3 paths, Fig. 5c). There were a series of researches reported that the mechanisms of PD were closed related to the acquired immune system [30], where CD8+ T cells killed SN dopamine neurons through the MHC class I complex [30]; and accumulative α-synuclein peptides (a risk PD factor) could be recognized by T cells [31]. In brief, the T cells contributed to the loss of dopamine neurons.

### The relationship between aging and PD were reflected by the aging-PD bipartite graphs

To study the relationships between the aging and PD markers, the coefficients were calculated based on each “aging-PD” pair. Then the results of PD and control samples were compared (as mentioned in Materials and Methods). The pair with the highest absolute differential correlation coefficient was GK2 and BCL11B. Evidences reported that GK2 was related to the mitochondrial LC-fatty acid beta-oxidation, where the efficient energy was produced. Energy metabolism was vital to life activities, and was dysregulated during accelerated aging thus in the context of neurodegenerative diseases [32, 33]. BCL11B was a fundamental transcriptional regulator in cell apoptosis, proliferation, and differentiation [34], thus related to the variation and development of neuronal subtypes of central nervous system [35]. Moreover, it was essential for the development of the immune system [34] and was considered as an interacting partner of T cells [36]. For example, the expression of cytokines (such as IL2, IL5 and IL13) in various T cells was depended on BCL11B [35, 36]. Therefore, BCL11B may be associated with aging and PD through the immune system.

The comprehending correlation mechanisms of aging and PD were further analyzed. As a result, GJC1 was identified the aging marker linking most PD markers and BCL11B was the PD marker linking to most aging markers (Figure S2). Strikingly, the importance of BCL11B was further highlighted. In addition, it has been reported that GJC1 was related to the ion channel activity. Therefore, the dysregulation of GJC1 during advanced aging was associated with ion imbanlances and the neuronal apoptosis [37], where the oxidation of ion channels was also a hallmark of cell deaths in PD progression [37]. In a word, the index of ion imbanlances was a bridge linking aging and PD.

In addtion, a sub-network composed of all the “aging-PD” shortest paths were picked out thereby, and these genes were ranked by their betweennesses (in descending order) along with the permutation *p*-values (shown in Table 4). There were 4 out of the top 10 genes whose p-values were significant. For example, BCL6 was with the largest betweeness 80 (the permutation p-value = 0), meaning that there were 80 shortest paths going through this gene. Obviously, BCL6 is a critical marker in cell apoptosis. It has also been reported that BCL6 contributed to the differentiation of follicular helper T cells and the inflammation activation of macrophages [38]. Therefore, it could be speculated that BCL6 was one of the most important markers linking accelerated aging and LOPD.

**Table 4:** The top 10 genes with the top betweenness in the aging acceleration network

Gene	Betweenness	*p*-value
BCL6	80	0*
ARPC5L	69	0.076
APBB1IP	39	0*
ASNS	23	0.081
BTN2A1	20	0.014*
CFLAR	15	0.003*
ANK3	11	0.082
CD2AP	11	0.195
ABHD3	9	0.340
CASP2	9	0.354

*:*p*-value< 0.05, significant

## Discussion

In this work, the relationship between aging and LOPD were studied through our computational pipeline. First, the aging markers were identified by the aging predictor. Thus, the improved PD predictor compared with the normal aging process was modeled to identify PD markers. Besides, the aging scores showed the aging acceleration pattern in LOPD compared with the control group. Further, the aging acceleration network based on aging scores was constructed, where the aging-PD paths revealed critical functions at system level.

**Fig. 6:**
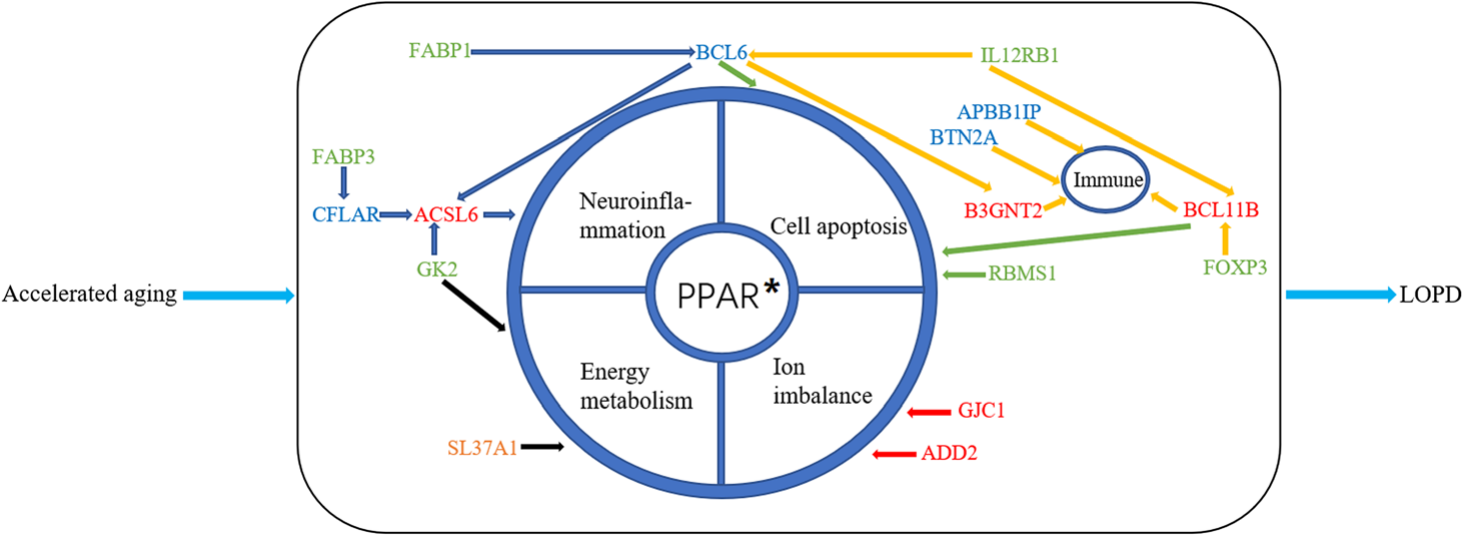
The mechanisms of accelerated aging triggering PD. The red genes are PD biomarkers, the green genes are aging biomarkers, the orange gene is the most important network node, and the blue genes are the top significant network nodes with the top highest betweenness. The blue arrows represent the links with PPAR signaling pathway, the black arrows represent the links with energy metabolism, the red arrows represent the links with ion imbalance, the green arrows represent the links with cell apoptosis, and the orange arrows represent the links with immune responses

The top aging marker (RBMS1) played a vital role in cell apoptosis [17]; the most notable PD risk biomarker (ADD2) revealed the importance of the mitochondrial Ca^2+^ imbanlance; and the most important network node SLC37A1 also indicated the mitochondria-related mechanisms of PD through the energy metabolism. Four out of top ten genes with the significant betweennesses suggested that the immune system was vulnerable to be dysregulated during advanced aging [39]. More importantly, the enrichment results highlighted the crucial core of the PPAR signaling pathway interacted with the neuroinflammation, mitochondrial oxidative stress and cell apoptosis. The most closely related “aging-PD” pair was GK2 and BCL11B, indicating the critical role of GK2 that was related to ATP production in mitochondria. BCL11B was also as the PD biomarker linked most aging biomarkers and associated to the immune response and eliminate inflammation. What’s more, GJC1 (as the aging marker linked the most PD markers) acted as a key role in the ion channel. In short, our results integrated aging and PD markers systematically (Fig. 6).

In the neuroimmune-endocrine theories of aging, the neuroinflammation response was exaggerated and prolonged in the healthy aged brain, thus advanced aging tended to induce a series of inflammatory factors amplified this response [40]. Our results further confirmed the importance of inflammation in the progression of PD [41]. In addition, apart from the mechanism of neuroinflammation-induced PD [42], other mechanisms of LOPD were integrated through our results. More interestingly, we proposed that the improved PD predictor was more reliable in exploring how PD was induced by aging acceleration. The PD markers identified by our improved prediction model have confirmed the dysfunctions in the mitochondrial Ca2+ pathway [43]. Further, the neurodegenerative disease was more vulnerable to be induced by excessive apoptosis in neuronal cells [44], thus the network markers with significant high betweenness also indicated the vital role of cell apoptosis, where the ion imbanlances also played an important role in PD progression (i.e. the function of GJC1). Strikingly, the enrichment results demonstrated that the PPAR signaling pathway played a central role in regulating the inflammation response, energy metabolisms and the cell apoptosis. In summary, our results integrated the neuronal apoptosis, neuroinflammations, mitochondrial metabolisms, and the ion imbanlances as comprehensive mechanisms of PD, where the PPAR signaling pathway may play a central role on the basis of immune disorders (Fig. 6).

In brief, our results indicated that LOPD was induced by accelerated aging, along with dysfunctions in the immune system [45]. In addition, the neuronal apoptosis, mitochondrial metabolic processes, the neuroinflammation and the ion imbanlances interacted with each other to induce PD coordinately. It has also been reported the homeostasis of these mechanisms would be disrupted during the brain aging, presenting the key risk factor for neurodegeneration [46]. Therefore, our results brought insight into a comprehensive understanding of LOPD development triggered accelerated aging.

## Conclusion

To conclude, the aging predictor and the improved PD predictor revealed potential risk biomarkers by machine learning methods, respectively. Then the aging scores were calculated to assess the aging process in both PD and control groups. The aging scores revealed significant aging acceleration in PD. Further, the aging acceleration network was constructed by comparing (partial) correlations of genes between LOPD and control samples. Additionally, the Aging-PD bipartite graph was utilized to investigate the relationship between aging and LOPD. As a result, the enrichment analysis indicated the critical mechanisms of LOPD during advanced aging. That is to say, during accelerated aging, the cell apoptosis, mitochondrial disorders, the ion imbanlances and the increased neuroinflammation combined to trigger PD coordinately, where the PPAR signaling pathway probably played the most critical role in immune dysfunctions.

## Materials and methods

### Gene expression profiles and data preprocessing

The gene expression profiles were obtained in Gene Expression Omnibus (GEO) database (https://www.ncbi.nlm.nih.gov/geo/), including GSE8397, GSE20295, GSE28894, GSE57475, GSE99039, and GSE15745 (Text S2). All of the selected PD or normal samples were not only with accurate age information, but also with more than 10 sample sizes. These samples were obtained from five different platforms: GPL96, GPL97, GPL570, GPL6104, and GPL6947.

The steps of obtaining gene expression profiles were as follows:

1. The probes of samples were converted to various gene symbols of platforms.
2. Gene expression values with the same gene symbols were summarized.
3. The total data matrix was integrated and the missing gene expression values were filled with 0.
4. The genes with zero expression values ≥30% were deleted.
5. Patients with early-onset PD (age < 50) were removed.

Finally, we collected the expression datasets of 425 healthy young samples (285 in training data and 140 in test data), 447 normal aged samples (300 + 147) and 392 PD samples (265 + 127) including 13,883 gene features (Text S1-S2).

In addition, the profiles in each platform was further normalized, seperately:

1. On account of the mean and the standard deviation of the normal samples, the z-score normalization was performed for both normal old samples and PD samples.
2. The Singular Value Decomposition (SVD) method was performed to eliminate the inter-sample variation based on the top three principal components of the normal and PD samples, respectively.
3. The z-score was then utilized to normalize all samples based on the mean and the standard deviation of the normal old samples.

### Modeling the aging predictor and identifying aging biomarkers

To model the reliable aging predictor, the normal samples were divided into training dataset and test dataset in which the ratio of training dataset samples to test dataset samples were close to 2:1. In addition, the number of young samples (< 50 years old) was similar to the number of old samples (≥50 years old) in both the training dataset and the test dataset. To evaluate the gene-age correlation, the Pearson correlation coefficient between genes and age labels was calculated:

1 $$ corr\left( gene, AgeGroup\right) $$

Then, 13,883 genes were sorted in descending order based on the absolute values of the correlation coefficients. Based on sorted gene symbols, k-NN (k = 5 with cosine distance) was performed to construct the top 100 aging predictors. The optimal model would be selected through the 10-fold cross-validation. To prove the predictability and efficiency of the aging predictor, the selected model was validated in the test dataset.

### Modeling the improved PD predictor and identifying PD biomarkers

To uncover the mechanism of LOPD in the aging acceleration process, normal young samples were considered as the auxiliary outlier group, and the improved PD predictor was modeled by comparing LOPD and normal aged samples (age ≥ 50). The deviations of PD and normal aged samples relative to healthy young samples were calculated as follows:

1. The first 3 principal components (row vectors) of healthy young samples (row vectors were samples, column vectors were genes) were extracted;
2. The original gene expression profiles of the normal aged and PD samples were substituted based on the residuals by regressing these 3 principal components of the healthy young samples, respectively.

Further, the new gene datasets from the normal aged and PD samples were divided into training dataset and test dataset. Similarly, the ratio of the training samples and the test samples was closed to 2:1, and the proportion of the normal old samples and PD samples was approximate in the training data and test data, respectively. The PD aging predictor was also constructed by sorting descending absolute values of Pearson correlation coefficients and utilizing k-NN (k = 5 with the cosine distance) method. The traditional PD predictor using original gene datasets was also calculated to compare and evaluate the prediction ability of the improved LOPD model.

### Calculating the aging scores

For all normal samples and PD samples, the quantitative aging scores were further summarized based on chronological age using the aging markers identified by kNN. The detailed pipeline was as follows:

1. The chronological age was transformed using the sigmoid function:
  2 $$ transformated\  age=\frac{1}{1+\exp \left(-\left( age-50\right)/50\right)} $$
2. The transformated age was predicted based on the aging markers using the linear regression:
  3 $$ transformated\  age=\sum {b}_i\ast aging\_ mar{\ker}_i $$

where *b*_*i*_ was the regression coefficient for each aging marker, respectively.

(3) The prediction of transformated age was used as the aging score;
(4) The Kruskal-Wallis test was used to compare the accerelated aging pattern between LOPD and the normal aged samples for different age groups.

### The construction of the aging acceleration network

The aging acceleration network based on the aging scores was constructed to further reveal the relationship between aging and PD. For the purpose of reliable network validation, the aging accelerated networks were constructed on the basis of training dataset and test dataset, respectively. First of all, for the respective 13,883 gene values of normal old samples and PD samples, the Pearson correlation coefficient as well as the partial correlation coefficient (based on the aging score) of any each pair of genes was calculated, respectively. Further, the assessment criteria of statistical significance was the Benjamini-Hochberg False Discovery Rates (FDR) < 0.05. Additionally, the absolute difference of partial correlation coefficient between PD samples and normal aged samples was calculated, as well as the absolute difference of correlation coefficient. If both of the absolute differences were greater than 0.5, the relationship between pairs of genes was further retained. Finally, with Fisher’s exact test, the similarity of two aging acceleration networks based on training dataset and test dataset would be tested. At the same time, scale-free property of the aging acceleration network was validated.

### The construction of the aging-PD bipartite graph

To better understand the detailed connections between aging biomarkers and PD biomarkers, the Aging-PD bipartite graphs were constructed in the context of the aging acceleration network, depending on the existed shortest paths (using the Dijkstra algorithm) from each aging marker to PD marker. To find out each pair of notable aging markers and PD markers, the absolute value of the difference between two correlation coefficients in the normal aged group and PD group was calculated, and the “aging-PD” pair with the opposite sign was further retained. Then the Aging-PD biomarker pair with the maximum absolute value should be noted. Moreover, the aging biomarker that most closely linked to PD markers and the PD biomarker that most closely linked to aging biomarkers were important. Therefore, for each aging marker, the sum of absolute values of PD markers was calculated. For each PD marker, the sum of absolute values of aging markers was also calculated. As a result, the aging/PD marker with high accumulated absolute differential correlations were the considered as critical genes interacted with PD/aging markers. As a result, totally 362 “aging-PD” pairs were identified out of 552 (69*8) pairs.

Further, the subnetwork with shortest pathways among selected “aging-PD” pairs was constructed, and genes in the subnetwork were sorted by their betweennesses in descending order. To test whether the top betweenness genes were hubs in the background network or not, we ran a permutation to count the occurrence times of the top genes in the shortest paths between random selected genes (containing the same numbers of “aging-PD” pairs, 69*8) when they were with greater betweennesses than those in our study. We repeated this process 1000 times, and the *p*-value was calculated as the proportion of occurrence times of the top betweenness genes in 1000 permutations.

### Enrichment analysis

To find vital biological functions, enrichment analysis was utilized. Gene Ontology (GO) terms and KEGG pathways were downloaded from Gene Set Enrichment Analysis (GSEA) platform (http://software.broadinstitute.org/gsea/downloads.jsp, version 7.0). The hypergeometric test was used to performed to estimate the enrichment of KEGG pathways or GO BP terms. The hypergeometric test formula was given as

4 $$ P\left(X\ge x\right)=1-\sum \limits_{k=0}^{x-1}\frac{C_M^k\ast {C}_{N-M}^{n-k}}{C_N^k} $$

where N is the total gene number of the gene sets, M shows the number of known genes (i.e. KEGG pathway, GO terms), n is the number of identified genes in each shortestpath, and k is the number of common genes between the known genes and the identified candidate genes (in each aging-PD shortest path). The p-value of each pathway was controlled by the Benjamini-Hochberg method. The final thresholds were *p* < 0.05 and FDR < 0.1.

## Supplementary information

**Additional file 1 **: **Figure S1.** The learning curve and ROC curve in the traditional PD predictor. **Figure S2.** The heatmap about the differential correlation coeffecients between the aging biomarkers and PD biomarkers. Xlable reprensents PD markers, Ylable represents aging markers.

**Additional file 2 **: **Text S1.** The gene set used in this work.

**Additional file 3 **: **Text S2.** The detailed datasets in this work.

**Additional file 4 **: **Table S1.** The top 69 aging biomarkers and their correlation coefficients. **Table S2.** The top 8 PD markers in the improved PD predictor and their correlation coefficients. **Table S3.** The top 94 PD markers in the traditional PD predictor and their correlation coefficients.

## Data Availability

The data supporting the results of this article are included and cited within the article and its additional files (i.e Text S1, Table S1 and Text S2).
