# Supplementary material for: The accelerated aging model reveals critical mechanisms of late-onset Parkinson’s disease
Source: BioData Min. 2020 Jun 10;13:4. doi: 10.1186/s13040-020-00215-w (PMC7288517; doi:10.1186/s13040-020-00215-w)
Supplement: Supplementary file 1 — Additional file 1 : Figure S1. The learning curve and ROC curve in the traditional PD predictor. Figure S2. The heatmap about the differential correlation coeffecients between the aging biomarkers and PD biomarkers. Xlable reprensents PD markers, Ylable represents aging markers. [file 13040_2020_215_MOESM1_ESM.docx]

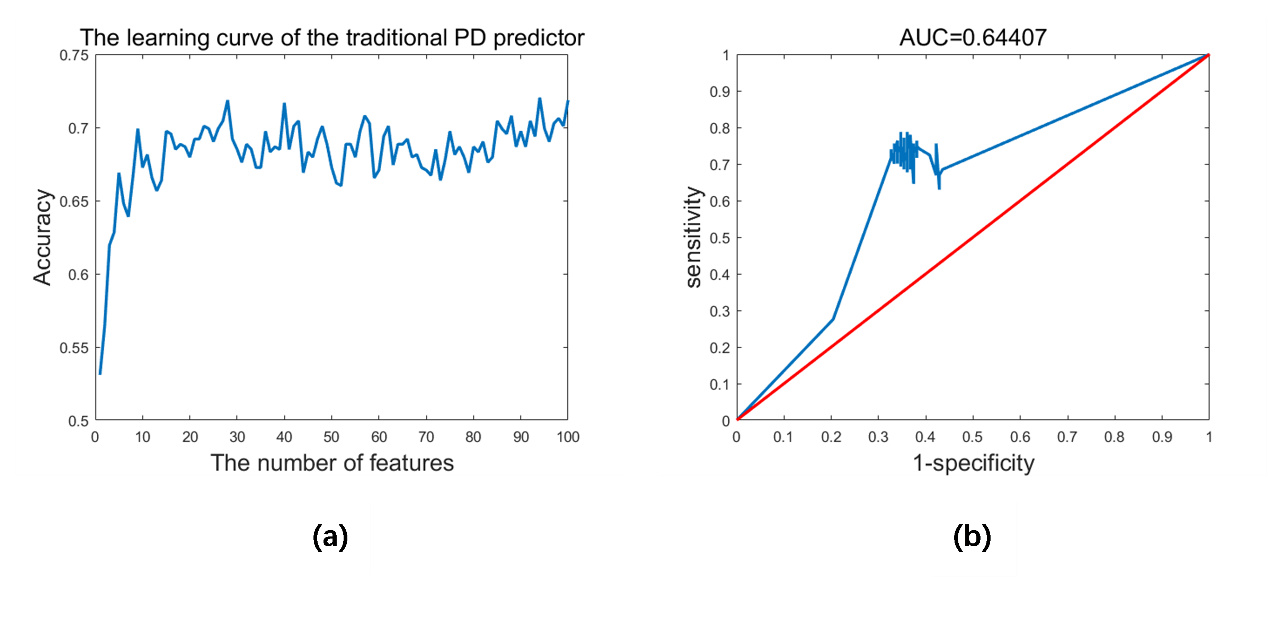


**Figure S1:** The learning curve and ROC curve in the traditional PD predictor.


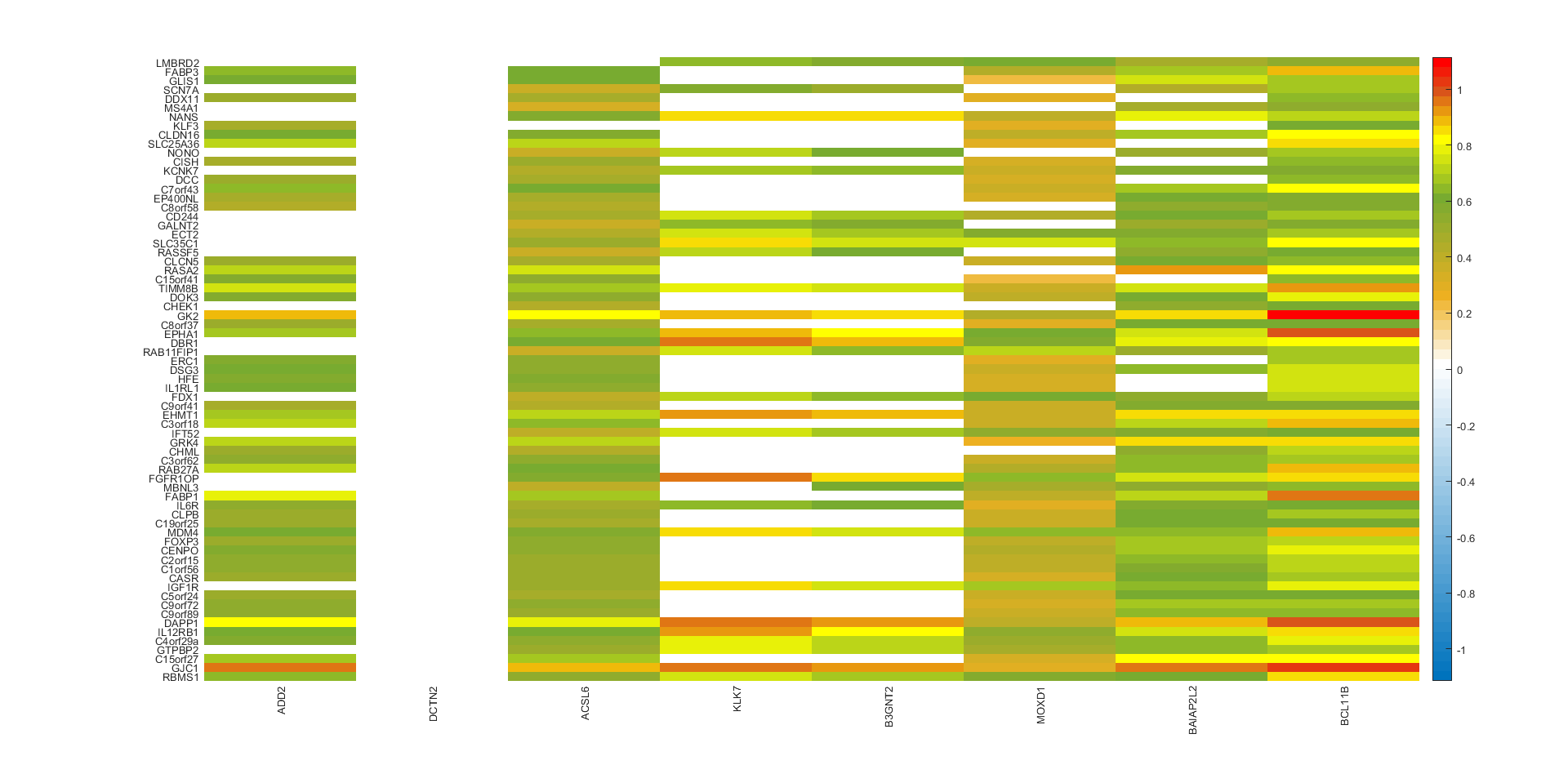


**Figure S2:** The heatmap about the differential correlation coeffecients between the aging biomarkers and PD biomarkers. Xlable reprensents PD biomarkers, Ylable represents aging biomarkers.
