# Supplementary material for: The accelerated aging model reveals critical mechanisms of late-onset Parkinson’s disease
Source: BioData Min. 2020 Jun 10;13:4. doi: 10.1186/s13040-020-00215-w (PMC7288517; doi:10.1186/s13040-020-00215-w)
Supplement: Supplementary file 4 — Additional file 4 : Table S1. The top 69 aging biomarkers and their correlation coefficients. Table S2. The top 8 PD markers in the improved PD predictor and their correlation coefficients. Table S3. The top 94 PD markers in the traditional PD predictor and their correlation coefficients. [file 13040_2020_215_MOESM4_ESM.doc]

**Table S1:** The top 69 aging biomarkers and their correlation coefficients

| Aging biomarkers | Correlation coefficients |
| --- | --- |
| RBMS1  GJC1  C15orf27  GTPBP2  C4orf29a  IL12RB1  DAPP1  C9orf89  C9orf72  C5orf24  IGF1R  CASR  C1orf56  C2orf15  CENPO  FOXP3  MDM4  C19orf25  CLPB  IL6R  FABP1  MBNL3  FGFR1OP  RAB27A  C3orf62  CHML  GRK4  IFT52  C3orf18  EHMT1  C9orf41  FDX1  IL1RL1  HFE  DSG3  ERC1  RAB11FIP1  DBR1  EPHA1  C8orf37  GK2  CHEK1  DOK3  TIMM8B  C15orf41  RASA2  CLCN5  RASSF5  SLC35C1  ECT2  GALNT2  CD244  C8orf58  EP400NL  C7orf43  DCC  KCNK7  CISH  NONO  SLC25A36  CLDN16  KLF3  NANS  MS4A1  DDX11  SCN7A  GLIS1  FABP3  LMBRD2 | 0.510810000000000  0.510630000000000  0.510060000000000  0.510020000000000  0.509930000000000  0.509720000000000  0.509610000000000  0.508520000000000  0.508140000000000  0.506940000000000  0.506650000000000  0.506610000000000  0.506560000000000  0.506530000000000  0.506520000000000  0.506430000000000  0.505850000000000  0.505820000000000  0.505680000000000  0.505610000000000  0.505540000000000  0.505540000000000  0.505530000000000  0.505470000000000  0.505340000000000  0.505300000000000  0.505130000000000  0.504960000000000  0.504830000000000  0.504830000000000  0.504640000000000  0.504530000000000  0.504450000000000  0.504100000000000  0.504060000000000  0.503850000000000  0.503790000000000  0.503580000000000  0.503460000000000  0.503410000000000  0.503050000000000  0.502990000000000  0.502980000000000  0.502790000000000  0.502670000000000  0.502280000000000  0.501490000000000  0.501280000000000  0.501280000000000  0.501020000000000  0.501000000000000  0.500800000000000  0.500780000000000  0.499820000000000  0.499700000000000  0.499700000000000  0.499670000000000  0.499510000000000  0.499480000000000  0.499200000000000  0.499090000000000  0.498960000000000  0.498790000000000  0.498690000000000  0.498680000000000  0.498630000000000  0.498560000000000  0.498420000000000  0.498400000000000 |

**Table S2:** The top 8 PD biomarkers in the improved PD predictor and their correlation coefficients

| The PD biomarkers of the improved PD predictor | Correlation coefficients |
| --- | --- |
| ADD2  DCTN2  ACSL6  KLK7  B3GNT2  MOXD1  BAIAP2L2  BCL11B | -0.143910000000000  -0.143330000000000  -0.142950000000000  -0.142840000000000  -0.142750000000000  -0.142670000000000  -0.142590000000000  -0.142550000000000 |

**Table S3:** The top 94 PD biomarkers in the traditional PD predictor and their correlation coefficients

| The PD biomarkers of the traditional PD predictor | Correlation coefficients |
| --- | --- |
| SLC37A1  USP13  MTRF1  ZDHHC3  CTPS2  FKBP14  NUP153  FNBP1  MARK3  SNIP1  DDX46  USP8  ZNF10  USP34  RBM15B  UXS1  TMEM57  TMEM50A  IK  VPS11  ZNF576  ZNF688  IPP  CYB5R4  NGLY1  SPATA5L1  TLK2  U2AF1  PSMD5  KIAA0907  ZXDB  ZBTB24  GIT2  ZNF195  NUP107  VPS53  ZNF614  SEC22A  RIOK2  PRUNE  FCF1  DIS3  SMEK1  KIDINS220  ZNF330  LRCH3  EDEM2  TAF2  LMAN1  LMBRD1  SF3A1  ZNF532  KLF13  TMEM11  UBE3C  ZNF35  IREB2  UTP18  ZNF143  PTEN  RLF  PELI2  GFRA3  THBS3  FBXL12  GTF3C5  THUMPD2  ZNF211  WIPI1  KLF6  SP2  GALNS  ZNF134  UBE2Q1  PDE6D  STARD8  MLH1  RINT1  TMEM39A  TFDP1  ZBED4  TRIM32  PPIA  EPB41L5  SNAP29  ZFP2  SHMT1  TTC27  PRPF38B  TXNDC11  ZNF180  GSR  TFCP2  TUBD1 | 0.467340000000000  0.462970000000000  0.460910000000000  0.456670000000000  0.455410000000000  0.454740000000000  0.454000000000000  0.453240000000000  0.452510000000000  0.449330000000000  0.449220000000000  0.448380000000000  0.447620000000000  0.446820000000000  0.444700000000000  0.443650000000000  0.443600000000000  0.442920000000000  0.442670000000000  0.442330000000000  0.441330000000000  0.440950000000000  0.440850000000000  0.440810000000000  0.440730000000000  0.440500000000000  0.440260000000000  0.439460000000000  0.439400000000000  0.438900000000000  0.438110000000000  0.437920000000000  0.437900000000000  0.437890000000000  0.437310000000000  0.437290000000000  0.437220000000000  0.437070000000000  0.436930000000000  0.436860000000000  0.436360000000000  0.436070000000000  0.435740000000000  0.435680000000000  0.435220000000000  0.435060000000000  0.434800000000000  0.434400000000000  0.434230000000000  0.433770000000000  0.433720000000000  0.433200000000000  0.432750000000000  0.432620000000000  0.432430000000000  0.432100000000000  0.431860000000000  0.431790000000000  0.431550000000000  0.431300000000000  0.431280000000000  0.431250000000000  0.431150000000000  0.430720000000000  0.430410000000000  0.430070000000000  0.430050000000000  0.429510000000000  0.429430000000000  0.429390000000000  0.428730000000000  0.428660000000000  0.428550000000000  0.428510000000000  0.428350000000000  0.428350000000000  0.427820000000000  0.427720000000000  0.427710000000000  0.427010000000000  0.426760000000000  0.426300000000000  0.426210000000000  0.426060000000000  0.425210000000000  0.425180000000000  0.425000000000000  0.424960000000000  0.424860000000000  0.424750000000000  0.424460000000000  0.424300000000000  0.424300000000000  0.424250000000000 |
